# Structural and Functional Brain Changes in Patients With Classic Trigeminal Neuralgia: A Combination of Voxel-Based Morphometry and Resting-State Functional MRI Study

**DOI:** 10.3389/fnins.2022.930765

**Published:** 2022-06-29

**Authors:** Hao Liu, Haiman Hou, Fangfang Li, Ruiping Zheng, Yong Zhang, Jingliang Cheng, Shaoqiang Han

**Affiliations:** ^1^Department of Magnetic Resonance Imaging, The First Affiliated Hospital of Zhengzhou University, Zhengzhou, China; ^2^Key Laboratory for Functional Magnetic Resonance Imaging and Molecular Imaging of Henan Province, Zhengzhou, China; ^3^Engineering Technology Research Center for Detection and Application of Brain Function of Henan Province, Zhengzhou, China; ^4^Engineering Research Center of Medical Imaging Intelligent Diagnosis and Treatment of Henan Province, Zhengzhou, China; ^5^Key Laboratory of Magnetic Resonance and Brain Function of Henan Province, Zhengzhou, China; ^6^Key Laboratory of Brain Function and Cognitive Magnetic Resonance Imaging of Zhengzhou, Zhengzhou, China; ^7^Key Laboratory of Imaging Intelligence Research medicine of Henan Province, Zhengzhou, China; ^8^Henan Engineering Research Center of Brain Function Development and Application, Zhengzhou, China; ^9^Department of Neurology, The First Affiliated Hospital of Zhengzhou University, Zhengzhou, China

**Keywords:** classic trigeminal neuralgia, voxel-based morphometry, amplitude of low-frequency fluctuations, resting-state functional MRI, neuropathic pain

## Abstract

**Objectives:**

Brain structural and functional abnormalities have been separately reported in patients with classic trigeminal neuralgia (CTN). However, whether and how the functional deficits are related to the structural alterations remains unclear. This study aims to investigate the anatomical and functional deficits in patients with CTN and explore their association.

**Methods:**

A total of 34 patients with CTN and 29 healthy controls (HCs) with age- and gender-matched were recruited. All subjects underwent structural and resting-state functional magnetic resonance imaging (fMRI) scanning and neuropsychological assessments. Voxel-based morphometry (VBM) was applied to characterize the alterations of gray matter volume (GMV). The amplitude of low-frequency fluctuation (ALFF) method was used to evaluate regional intrinsic spontaneous neural activity. Further correlation analyses were performed between the structural and functional changes and neuropsychological assessments.

**Results:**

Compared to the HCs, significantly reduced GMV was revealed in the right hippocampus, right fusiform gyrus (FFG), and temporal-parietal regions (the left superior/middle temporal gyrus, left operculo-insular gyrus, left inferior parietal lobule, and right inferior temporal gyrus) in patients with CTN. Increased functional activity measured by zALFF was observed mainly in the limbic system (the bilateral hippocampus and bilateral parahippocampal gyrus), bilateral FFG, basal ganglia system (the bilateral putamen, bilateral caudate, and right pallidum), left thalamus, left cerebellum, midbrain, and pons. Moreover, the right hippocampus and FFG were the overlapped regions with both functional and anatomical deficits. Furthermore, GMV in the right hippocampus was negatively correlated with pain intensity, anxiety, and depression. GMV in the right FFG was negatively correlated with illness duration. The zALFF value in the right FFG was positively correlated with anxiety.

**Conclusion:**

Our results revealed concurrent structural and functional changes in patients with CTN, indicating that the CTN is a brain disorder with structural and functional abnormalities. Moreover, the overlapping structural and functional changes in the right hippocampus and FFG suggested that anatomical and functional changes might alter dependently in patients with CTN. These findings highlight the vital role of hippocampus and FFG in the pathophysiology of CTN.

## Introduction

Classic trigeminal neuralgia (CTN) is a chronic neuropathic pain that is limited to one or more branches of the trigeminal nerve (Scholz et al., 2019). It usually presents as abrupt paroxysmal electric shock-like or stabbing pain and can be provoked by normally innocuous mechanical stimuli or occurs spontaneously. Unlike other neuropathic pain conditions, pain attacks become more frequent with disease progression and may become sustained subsequently (Headache Classification Committee of the International Headache Society, 2013), which would seriously affect the patient’s physical and mental health (DeSouza et al., 2016). The prevailing theory of CTN etiology is neurovascular compression at the entry zone of a nerve root (Nurmikko and Eldridge, 2001; Maarbjerg et al., 2015). However, till now, the pathophysiology of CTN is still unclear. Recently, several neuroimaging studies have found brain structural and functional abnormalities in patients with CTN (Obermann et al., 2013; DeSouza et al., 2016; Wang et al., 2017a; Tsai et al., 2018; Tang et al., 2020).

Several brain structural studies have revealed gray matter volume (GMV) changes in patients with CTN which mainly involved primary somatosensory cortex, insula, thalamus, anterior cingulate cortex, basal ganglia, hippocampus, temporal cortex, and cerebellum, most notably pain matrix (Obermann et al., 2013; Wang et al., 2017a; Tsai et al., 2018; Tang et al., 2020; Wu et al., 2020). Meanwhile, brain functional abnormalities revealed by functional magnetic resonance imaging (fMRI) studies in patients with CTN have been detected and mainly localized in the prefrontal, temporal, and parietal regions, posterior cingulate cortex, insula, and cerebellum, particularly in the salient network and default-mode network (Wang et al., 2017b; Chen et al., 2019; Tsai et al., 2019; Zhang et al., 2021). It can be observed that there were different and overlapped brain regions, where structural and functional abnormalities were identified in the above previous studies. It is well-known that brain structure and function are intimately related to each other. Thereby, altered brain function would probably result in altered gray matter changes or vice versa. However, most of these studies have only investigated functional or anatomical changes alone. Few studies have focused on structural and functional changes in the same sample; thereby, it is not clear whether concurrent structural and functional abnormalities could exist or not. A more important issue that remains unclear is whether and how the functional deficits are related to the anatomical alterations. If overlapping regions with structural and functional alterations were observed, it may indicate that the structure and function of those regions alter simultaneously in patients with CTN. The significance of defining concurrent structural and functional deficits may provide specific insights to unravel adaptive or maladaptive changes occurring in brain regions in patients with CTN.

Therefore, we used a multimodal neuroimaging approach to investigate the anatomical and functional alterations in CTN. Voxel-based morphometry (VBM) was used to analyze GMV changes, and the amplitude of low-frequency fluctuation (ALFF) method was applied to evaluate the regional spontaneous brain activity alterations. The aim of this study was to characterize the changes in GMV and ALFF in patients with CTN, the relationship between the functional alterations and anatomical deficits, as well as their association with clinical variables in patients with CTN. According to the current understanding of CTN, we hypothesized that concurrent structural and functional changes would be present in patients with CTN, and there would be overlapped regions with both functional and anatomical alterations.

## Methods

### Participants

A total of 34 patients with CTN were recruited prospectively from the First Affiliated Hospital of Zhengzhou University. The diagnostic criteria of CTN are based on the International Classification of Headache Disorders (ICHD-3) (Headache Classification Committee of the International Headache Society, 2013). Two neurologists confirmed the diagnosis according to the ICHD-3. The inclusion criteria for the patients were as follows: (i) age > 18 years; (ii) right-hand dominance; (iii) unilateral pain restricted to one or more branches of the trigeminal nerve branches; (iv) intense, shooting or stabbing, and abrupt pain paroxysms, with or without the trigger of normally innocuous mechanical stimuli or orofacial movements; (v) no other neurological or sensory deficits; and (vi) no evident abnormal lesions on conventional T2-weighted image. The exclusion criteria for the patients were as follows: (i) any other causes of facial pain; (ii) other primary headache disorders; (iii) neural-associated or psychiatric disorders; (iv) history of brain surgery; and (v) contraindications to MRI scan.

A total of 29 healthy controls (HCs) with age- and gender-matched were also recruited for this study. Inclusion criteria for HC were as follows: (i) age > 18 years; (ii) right-hand dominance; (iii) no neuropsychiatric disorders; and (iv) no contraindications to MRI scan.

This study was approved by the Ethics Committee of The First Affiliated Hospital of Zhengzhou University. According to the Declaration of Helsinki, written consent was obtained from each participant.

### Questionnaires and Ratings

The equations should be inserted in editable format from the equation editor. The visual analog scale (VAS) ranging from 0 (no pain) to 10 (the worst pain) was used to grade the pain intensity of patients with CTN. Patients were required to rate their pain intensity in the last 7 days using the VAS, and then the average score was calculated. The Hamilton Depression Rating Scale (HAMD) and the Hamilton Anxiety Rating Scale (HAMA) were applied to evaluate the anxious and depressive symptoms. All questionnaire assessment was performed under the supervision of experimenters.

### MRI Data Acquisition

MRI data were collected using a 3.0-T scanner (Discovery 750 System, Milwaukee, WI, United States), equipped with a 16-channel phased-array head coil. All participants were instructed to stay awake and be relaxed but to keep their eyes closed without falling asleep during scanning. In order to reduce the noise and avoid head motion, the earplugs and foam pads were used. First, a T2-weighted imaging sequence was acquired in all participants to rule out the possibility of asymptomatic lesions. Then, a high-resolution structural image was acquired using the following parameters: field of view (FOV) = 256 × 256 mm^2^, matrix = 256 × 256, time of repetition (TR) = 8.15 ms, time of inversion = 450 ms, time of echo (TE) = 3.17 ms, spatial resolution = 1.00 × 1.00 mm^2^, and flip angle = 12.0*^circ^*. The rs-fMRI data were obtained using the following parameters: 32 contiguous slices, FOV = 220 × 220 mm^2^, in-plane matrix = 64 × 64, spatial resolution = 3.44 × 3.44 × 4 mm^3^, *TR* = 2,000 ms, *TE* = 30 ms, flip angle = 90*^circ^*, and a total of 180 volumes and lasted 360 s for each subject.

### Voxel-Based Morphometry Analysis

Voxel-based morphometry analysis was performed following the standard pipeline of the CAT 12 toolbox^1^ to obtain GMVs. The details could be referred to Ashburner’s (2009) study. The main steps were as follows: bias-field correction, segmentation (gray matter, white matter, and cerebrospinal fluid), adjustment for partial volume effects, normalization into Montreal Neurological Institute space, resampled to 1.5 mm × 1.5 mm × 1.5 mm, and non-linear modulation (Ashburner, 2009; Han et al., 2022). Finally, the gray matter maps were smoothed using a 6-mm full width at half maximum (FWHM) Gaussian kernel. The total intracranial volume for each participant was calculated for the next process (Han et al., 2021a,b).

### Resting-Functional Magnetic Resonance Imaging Data Preprocessing and Calculation of zALFF

The resting-fMRI data were preprocessed using Data Processing Assistant for Resting-State fMRI package (DPARSFA).^2^ The first 10 volumes were deleted and then the remaining functional images were corrected for slice timing and realignment. The mean frame-wise displacement (FD) was calculated for each subject according to a previously published formula (Power et al., 2012; Han et al., 2020). Subjects were excluded if the translational and rotational displacement exceeded 3.0 mm or 3.0° from subsequent analyses. The functional images were spatially normalized to the standard EPI template and resampled to 3 × 3 × 3 mm^3^. Subsequently, the functional images were further smoothed with 6 × 6 × 6 mm^3^ FWHM Gaussian kernel and detrended to reduce low-frequency drift. Next, several nuisance covariates, including white matter signals, cerebrospinal fluid signals, and Friston-24 head motion parameters (Satterthwaite et al., 2012), were regressed out. Then, temporal band-pass filter (0.01–0.08 Hz) was conducted. Scrubbing with cubic spline interpolation was used to exclude the influence of head motion and ensure the contiguous time points.

The ALFF maps were calculated using the resting-state fMRI data processing toolbox (REST) (Song et al., 2011) and zALFF maps (subtracting the global mean value and dividing it by the standard deviation) were chosen for the following analysis.

### Statistical Analysis

The SPSS software version 23.0 (IBM Corporation, Armonk, NY, United States) was used for the demographic and clinical data analyses. Two-sample *t*-tests (for age, HAMA, and HAMD scores) and chi-square tests (for gender) were used to analyze the differences between patients with CTN and HCs subjects. To further investigate the changes in GMV and zALFF, a two-sample *t*-test was performed between the patients with CTN and HCs, with age, gender, and mean FD as covariates using SPM12. Multiple comparisons were corrected by false discovery rate (FDR) approach (*p* < 0.05).

To verify the overlapped brain regions between functional and anatomical alterations in the CTN, the regions with significant abnormalities in GMV and zALFF were overlaid on the same template, as described in the previous studies (Ren et al., 2013; Guo et al., 2015).

### Correlation Analysis With Clinical Variables

Once the overlapped regions with significant differences in GMV and zALFF were found, the GMV and zALFF values from those brain regions were extracted, respectively. The Pearson correlation was performed to examine the relationship between the mean GMV values and the mean zALFF values in the overlapped regions. Then, a two-tailed partial correlation analysis was performed to further evaluate the relationship between the values (GMV and zALFF) and clinical variables (VAS scores, disease duration, HAMD, and HAMA scores) in the CTN group, controlling for age and gender. A statistically significant threshold of *p* < 0.05 was set for all correlation analyses.

## Results

### Demographic and Clinical Characteristics

The demographics and clinical features of the two groups are summarized in Table 1. No significant differences were found between patients with CTN and HC subjects in terms of demographic characteristics, such as age and gender. Compared with HCs, patients with CTN had significantly higher scores on the HAMA and HAMD.

**TABLE 1:** Demographic and clinical characteristics.

Variables	CTN (*n* = 34)	HC (*n* = 29)	*t*/χ^2^	*P*-value
Age (years)	53.06 ± 10.91	54.21 ± 6.33	–0.520	0.606
Gender (male/female)	16/18	15/14	0.136	0.712
Duration of CTN (years)	4.63 ± 3.53	NA		
VAS	7.97 ± 1.42	NA		
Side affected (L/R), n	14/20	NA		
Score of HAMA	8.56 ± 6.01	3.93 ± 2.92	3.98	0.001
Score of HAMD	10.62 ± 6.73	4.6 ± 2.27	4.88	0.001

*CTN, classic trigeminal neuralgia; HC, healthy control; VAS, Visual Analog Scale; HAMA, Hamilton Anxiety Rating Scale; HAMD, Hamilton Depression Rating Scale; NA, not applicable.*

### Gray Matter Volume Changes

**FIGURE 1:**
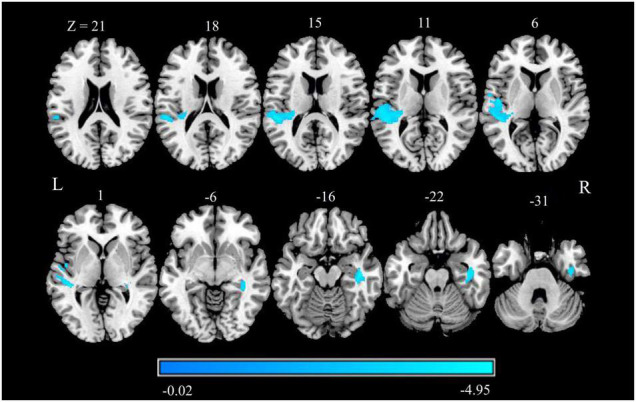
GMV alterations in patients with CTN. Differences between the patients with CTN and HCs were analyzed using a two-sample *t*-test. The statistical significance level was set at *p* < 0.05, FDR corrected. Patients with CTN showed significantly increased GMV in the right hippocampus, right FFG, right ITG, left STG, left operculo-insular gyrus, and left MTG. The color bar displayed *t*-values. GMV, gray matter volume; CTN, classic trigeminal neuralgia; HC, healthy control; FFG, fusiform gyrus; ITG, inferior temporal gyrus; STG, superior temporal gyrus; MTG, middle temporal gyrus.

Compared with HCs, patients with CTN exhibited significantly reduced GMV in the right hippocampus, right fusiform gyrus (FFG), right inferior temporal gyrus (ITG), left superior temporal gyrus (STG), left operculo-insular gyrus, and left middle temporal gyrus (MTG) (Figure 1 and Table 2) (*P* < 0.05, FDR corrected).

**TABLE 2:** Brain regions displaying significant differences in GMV between patients with CTN and HC subjects.

Anatomical region	MNI coordinates			Cluster size	*T*-value
			
	*x*	*y*	*z*		
Fusiform_R	41	–18	–26	315	4.67
Hippocampus_R	41	–30	–12	273	4.78
Inferior temporal gyrus_R	43	–19	–19	150	3.69
Superior temporal gyrus_L	–48	–35	15	1457	4.60
Operculo-insular_L	–37	–30	12	163	3.99

*GMV, gray matter volume; CTN, classic trigeminal neuralgia; HC, healthy control; MNI, Montreal Neurological Institute; L, left; R, right.*

### zALFF Changes

**FIGURE 2:**
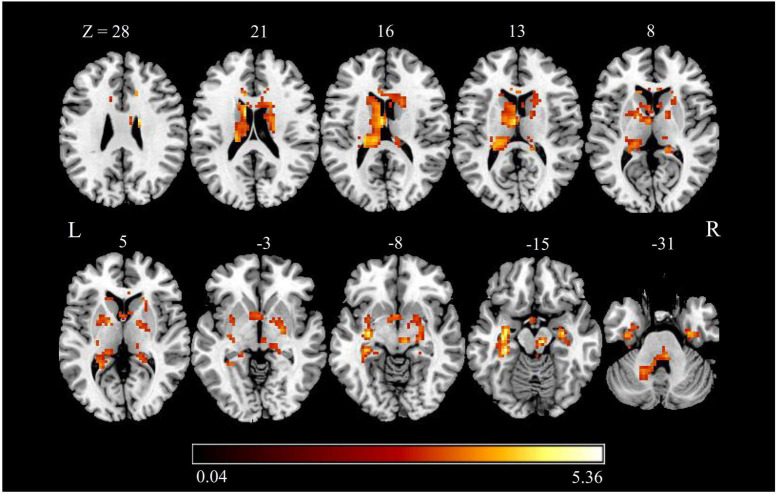
zALFF alterations in patients with CTN. The differences between the CTN patients and HCs were analyzed using a two-sample *t*-test. The statistical significance level was set at *p* < 0.05, FDR corrected. Patients with CTN showed significantly decreased zALFF in the bilateral hippocampus, bilateral fusiform FFG, bilateral caudate, left thalamus, bilateral putamen, right pallidum, bilateral PHG, and left cerebellum. The color bar displayed *t*-values. ALFF, amplitude of low-frequency fluctuation; CTN, classic trigeminal neuralgia; HC, healthy control; FFG, fusiform gyrus; PHG, parahippocampal gyrus.

Brain regions with significant zALFF differences are shown in Figure 2 and Table 3. Compared with HCs, patients with CTN showed significantly increased zALFF in the bilateral hippocampus, bilateral FFG, bilateral caudate, left thalamus, bilateral putamen, right pallidum, bilateral parahippocampal gyrus (PHG), left cerebellum, midbrain, and pons (*P* < 0.05, FDR corrected).

**TABLE 3:** Brain regions with altered zALFF in patients with CTN.

Regions	MNI coordinates			Voxels	*T*-value
			
	*x*	*y*	*z*		
Hippocampus_R	33	–15	–21	50	5.09
Fusiform_R	34	–10	–32	41	3.27
Caudate_R	10	7	1	64	3.01
Pallidum_R	17	–2	0	44	3.48
Fusiform_L	–33	–21	–22	117	4.19
Hippocampus_L	–33	–18	–18	100	5.20
Putamen_L	–24	3	3	49	3.32
Thalamus_L	–17	–28	10	79	3.24
Caudate_L	–6	5	10	101	3.17
Cerebellum Anterior Lobe	–18	–51	–33	85	4.14
ParaHippocampal_R	33	–18	–24	41	4.66
ParaHippocampal_L	–30	–30	–15	32	5.38

*ALFF, amplitude of low-frequency fluctuation; CTN, classic trigeminal neuralgia; HC, healthy control; MNI, Montreal Neurological Institute; L, left; R, right.*

### Association Between Functional and Anatomical Findings

**FIGURE 3:**
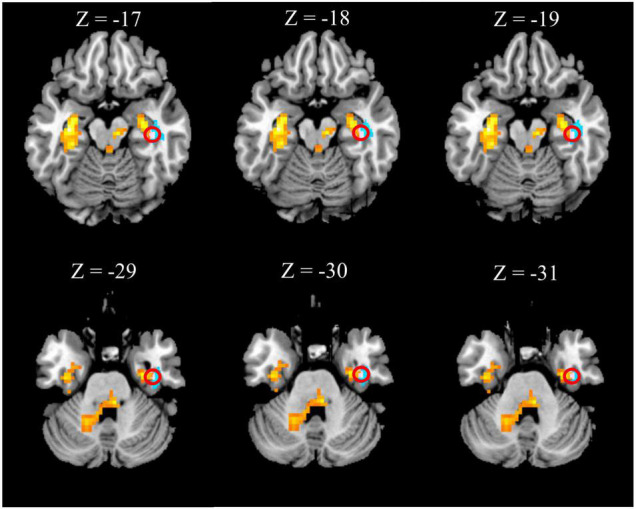
Overlapped brain region with structural and functional changes. The right hippocampus (first row) and right fusiform gyrus (second row) within the red circle represent the overlapped areas with both increased zALFF (hot colored regions) and decreased GMV (cold colored regions) in patients with CTN. GMV, gray matter volume; ALFF, amplitude of low-frequency fluctuation; CTN, classic trigeminal neuralgia.

**FIGURE 4:**
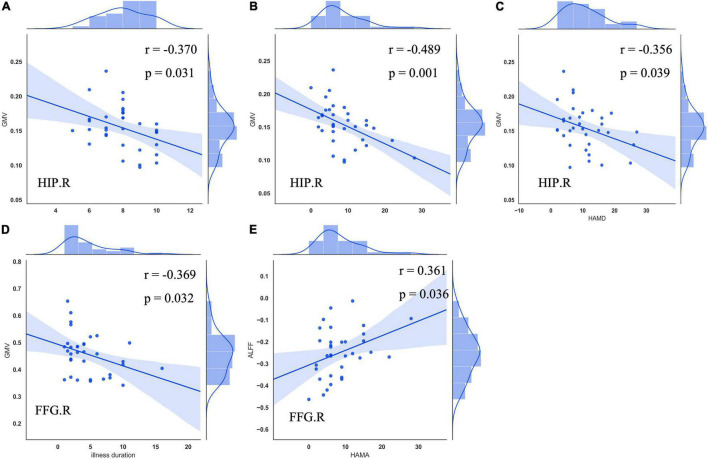
Correlation between GMV, zALFF alterations, and clinical variables in patients with CTN. GMV in the right hippocampus was negatively associated with VAS **(A)**, HAMA **(B)**, and HAMD **(C)** scores of patients with CTN. GMV in the right FFG was negatively correlated with illness duration **(D)**. Increased zALFF in the right FFG was positively correlated with HAMA score of the patients with CTN **(E)**. GMV, gray matter volume; ALFF, amplitude of low-frequency fluctuation; CTN, classic trigeminal neuralgia; HIP, hippocampus; VAS, visual analog scale; HAMA, Hamilton Anxiety Rating Scale; HAMD, Hamilton Depression Rating Scale; FFG, fusiform gyrus.

The right hippocampus and right FFG were overlapped regions with both functional and anatomical abnormalities in the patients with CTN (Figure 3). No significant correlation between the GMV and zALFF values in those overlapped regions was found. Furthermore, GMV in the right hippocampus was negatively correlated with pain intensity (*r* = –0.370, *P* = 0.031), anxiety (*r* = –0.489, *P* = 0.001), and depression scores (*r* = -0.356, *P* = 0.039) of the patients with CTN. GMV in the right FFG was negatively associated with the illness duration (*r* = –0.369, *P* = 0.032). There was a positive correlation of the zALFF value in the right FFG with anxiety (*r* = 0.361, *P* = 0.036) scores of the patients with CTN (Figure 4).

## Discussion

This study revealed concurrent brain anatomical and functional abnormalities in patients with CTN using the VBM and ALFF methods. Reductions in GMV were found mainly in the temporal-parietal regions (the left superior/middle temporal gyrus, left operculo-insular gyrus, left inferior parietal lobule, right ITG, right FFG) and limbic system (the right hippocampus). The increased regional spontaneous functional activities were observed in the basal ganglia system (the bilateral putamen, bilateral caudate, and right pallidum), limbic system (the bilateral hippocampus and bilateral PHG), left thalamus, bilateral FFG, left cerebellum, midbrain, and pons. More importantly, the right hippocampus and right FFG were the overlapped regions with both functional and anatomical alterations, suggesting that the structure and function of the brain might alter synchronously in CTN. GMV reduction in the right hippocampus was negatively related to the pain intensity and negative emotions. GMV reduction in the right FFG was negatively related to the illness duration. Increased activity in the right FFG was positively correlated with anxiety.

Our study identified reduced GMV in multiple brain regions, including ITG, STG, MTG, left operculo-insular cortex, and inferior parietal lobule. The GMV atrophy in these brain regions may indicate neural damage and loss during the recurrent attack of pain in patients with CTN. This may represent the cortical maladaptive response to the long-term and repeated nociceptive input and pain modulation. It is notable that most of the brain regions with reduced GMV are located in the temporal gyrus, which is consistent with previous studies (Wang et al., 2017a,^2019^; Wu et al., 2020), indicating that the temporal lobe was particularly vulnerable in CTN. A possible explanation is that multiple temporal regions are involved in the modulation of the emotional and motivational aspects of pain. The STG has been involved in the perception of emotions, and MTG is the component of the default mode network and served as the key part of the dynamic pain connectome (Liotti et al., 2000; Smallwood et al., 2013; Kucyi et al., 2014). The ITG has the projection to the amygdala and hippocampus, which are related to the process of emotion and cognition (Webster et al., 1991; Vincent and Hadjikhani, 2007). The potential mechanism is that the STG, MTG, ITG, and hippocampus were recruited to regulate aversive memory recognition and maintain the emotions in the modulation and anticipation of severe pain (Price, 2000; Tillfors et al., 2002; Bauch et al., 2014). After a prolonged pain attack, these responses would probably become maladaptive, lead to allostatic load over time, and cause negative emotional disorders. Therefore, we speculate that the GMV atrophy of temporal regions results in impairment of emotional processing aspect of pain and contributes to the negative moods observed in patients with CTN.

Meanwhile, compared to HCs, increased spontaneous brain activity was identified mainly in subcortical nuclei and limbic system in the CTN group. Several previous studies have also reported an increased spontaneous functional activity, but the involved brain regions varied among the studies, including temporal gyrus, occipital gyrus, precentral gyrus, and cerebellum (Chen et al., 2019; Xiang et al., 2019). The putamen, caudate, pallidum, thalamus, and midbrain are the main nodes of the cortex-basal ganglia-thalamus loops, which are involved in motor control. This loop has been reported that could regulate the motor and behavioral response to pain (Bingel et al., 2004; Starr et al., 2011). Thereby, the hyperactivity in the cortex-basal ganglia-thalamus loops and limbic system revealed in our study may be a compensatory response to pain attacks. It is reasonable to assume that abnormal hyperactivity of this loop could prompt patients to limit their orofacial movements, such as chewing, in order to avoid triggering pain (Bennetto et al., 2007; Zhang et al., 2018). Besides, central sensitization, which has been reported in patients with chronic daily headache (Chen et al., 2011; Maleki et al., 2012), may be another possible explanation for the increased spontaneous functional activity in our study. Central sensitization would cause the subcortical nuclei and limbic system to be easily activated and present as increased spontaneous functional activity. Furthermore, in previous studies, the altered GMV of basal ganglia and thalamus in patients with CTN has been reported (Gustin et al., 2011; Desouza et al., 2013; Zhong et al., 2018; Henssen et al., 2019), which proved that basal ganglia and thalamus were involved in the pathogenesis of CTN. However, we did not observe GMV alterations in basal ganglia and thalamus in patients with CTN, which were caused by the different disease durations of CTN in our study. This is supported by Shen et al.’s (2022) study, which used VBM to investigate the changes in brain structure in patients with CTN grouped according to disease duration and they found GMV alterations in different brain regions at different stages of CTN. Tsai et al. (2018) found reduced volume in the thalamus, but along with the duration of the disease, the volume of the thalamus gradually increased. Those findings suggest that the GMV alterations are highly dynamic. Longitudinal neuroimaging investigations in patients with CTN with analysis of both structure and function would provide further evidence for the dynamic changes in pain disorders.

More importantly, our findings revealed that the right hippocampus and FFG were the overlapped regions with both reduced GMV and increased zALFF, suggesting that the functional and anatomical alterations might alter simultaneously and dependently. Consistent with our study, reduced GMV in the hippocampus has also been reported in other studies of CTN (Hayes et al., 2017; Wang et al., 2019), but they did not investigate the functional change in the hippocampus. In the correlation analysis, we found that the volume of right FFG was negatively associated with the duration of CTN. Vaculik et al. (2019) reported a similar finding that hippocampal volume was negatively correlated with the illness duration in patients with CTN. These findings indicated that the volume of the hippocampus and FFG would gradually decrease along with the duration of the disease. The anatomical alterations revealed by VBM may reflect more stable and long-standing abnormalities. The reduced GMV of the hippocampus and FFG in patients with CTN may represent maladaptive plasticity caused by the prolonged pain attack. It is unexpected that the regional spontaneous activities in those GMV-reduced regions were found to increase concurrently in our study. Functional alterations measured by the zALFF method may represent physiological compensatory changes during the recurrent acute pain attacks. Although the volume of the right hippocampus and FFG was found to be reduced, we considered that the degree of reduction in these regions may not be sufficient yet to cause decreased functional activity, and these regions were still capable of exhibiting compensatory response to the recurring pain attacks in patients with CTN. Therefore, we speculate that the increased functional activity detected in our study probably was an adaptive and compensatory response of the brain to meet the increased demand for pain processing during the recurring attacks of CTN. The other possible explanations of these functional–structural alterations are that GMV loss may be a result of increased functional activity. Malenka (2003) found that alterations in neuronal activity can elicit long-lasting changes in the synaptic function and dendritic spine density, which may consequently lead to gray matter changes (McEwen, 1999; Klausberger, 2009; Metz et al., 2009). Similar to our findings, Drevets et al. (1997) reported decreased volume and increased activity in subgenual prefrontal cortex in patients with major depression. Taken together, our findings suggested that the functional changes are accompanied by anatomical alterations in patients with CTN.

The hippocampus, an important component of the limbic system, has widespread anatomical connections to cortical and subcortical regions and has key roles in learning, memory, cognition, and emotion formation (Fortin et al., 2002; Nestler et al., 2002; Kim et al., 2015). Accumulating evidence demonstrated that the hippocampus participates in pain processing, especially for affective and motivational component of pain (Liu and Chen, 2009; Mutso et al., 2014; Gerrits et al., 2015; Apkarian et al., 2016). It has been speculated that the hippocampal formation amplifies aversive effects as a protective mechanism to define appropriate behavioral responses (Maleki et al., 2013). Longitudinal imaging studies suggest that the representation of pain gradually shifts from sensory to emotional circuits and limbic structures as the pain disorders progress and become chronic (Hashmi et al., 2013; McCarberg and Peppin, 2019). Previous studies have reported that patients with CTN have an increased risk of developing psychiatric comorbidities, including anxiety and depression (Wu et al., 2015). Meanwhile, the GMV in the right hippocampus was found to be negatively related to the severity of pain, anxiety, and depression scores in our study. This suggested that the degree of GMV abnormalities in the right hippocampus may reflect pain severity and be related to the negative emotions in patients with CTN. In patients with migraine, morphological, and functional alterations in the hippocampus have also been reported, and these changes were associated with headache frequency, accumulative number of migraine attacks, and severity of anxiety and depression (Liu et al., 2018). Combined with our findings, it is strongly suggested that the hippocampus participated in the pathophysiological process of neuropathic pain disorders, especially in the affective and cognitive dimension of pain, which mainly contribute to the pain chronification.

It is interesting that the right FFG have similar anatomical and functional changes to the right hippocampus. Several studies confirmed cortical atrophy in the FFG in patients with CTN (Parise et al., 2014; Li et al., 2017). Chen et al. (2019) reported increased ALFF in the bilateral fusiform cortex. Painful electrical shock has been found to activate the fusiform cortex (Ter Minassian et al., 2013). Thus, it is speculated that pain in CTN could also activate FFG, as the feature of pain in CTN is electrical-shock-like pain. The potential mechanism may be that the fusiform cortex relates to the retrieval of similar sensation in patients with CTN during recurrent electrical-shock-like pain. Besides, the fusiform cortex was related to the retrieval of similar sensation in patients with CTN during recurrent electrical-shock-like pain. Moreover, the regional spontaneous activity of the right FFG was positively correlated with anxiety in our study, indicating that the FFG was related to the modulation of affective aspect of pain. Therefore, the present findings added robust evidence that FFG is involved in the pain perception and modulation in CTN.

### Limitations

In this study, there are several limitations that should be addressed. First, we cannot exclude the potential effect of anti-epileptic agents on brain structure and function. Thus, this would be an important confounder of the study. Second, this study was a cross-sectional study with a relatively small sample size, so the structural and functional findings here were exploratory and preliminary. Future longitudinal studies with a large number of patients with CTN should be recruited to further clarify the relationship between the structural and functional alterations. In addition, the current analysis focused on regional intrinsic neuronal activity, and the effects of the interactions between different brain areas in patients with CTN need to be analyzed.

## Conclusion

Our results present evidence that concurrent anatomical and functional alterations occurred in the patients with CTN, indicating that the CTN is a brain disorder with structural and functional abnormalities. The overlapping structural and functional changes in the right hippocampus and FFG of CTN suggested that anatomical and functional changes might alter dependently in patients with CTN, and suggested the vital role of hippocampus and FFG in the pathophysiology of CTN. Therefore, this study highlights the importance of combining structural and functional MRI methods, which could offer complementary information for the understanding of the pathophysiology and chronification of CTN. Future studies should illustrate the causes of functional and anatomical changes and clarify whether the structural and functional alterations can be reversed after the effective treatment of CTN.

## Data Availability Statement

The raw data supporting the conclusions of this article will be made available by the authors, without undue reservation.

## Ethics Statement

The studies involving human participants were reviewed and approved by Ethics Committee of the First Affiliated Hospital of Zhengzhou University. The patients/participants provided their written informed consent to participate in this study.

## Author Contributions

HL, JC, and SH conceived and designed the study. HL and FL supervised the conduct of the study. HL, RZ, and YZ contributed to the MR data acquisition. FL and HH contributed to the clinical data acquisition. HL and HH analyzed the data and drafted the original manuscript writing. HL and SH contributed to the methodology and software and data curation. HH, YZ, and SH reviewed and revised the manuscript. All authors read and approved the final manuscript.

## Conflict of Interest

The authors declare that the research was conducted in the absence of any commercial or financial relationships that could be construed as a potential conflict of interest.

## Publisher’s Note

All claims expressed in this article are solely those of the authors and do not necessarily represent those of their affiliated organizations, or those of the publisher, the editors and the reviewers. Any product that may be evaluated in this article, or claim that may be made by its manufacturer, is not guaranteed or endorsed by the publisher.

## Footnotes

1
http://dbm.neuro.uni-jena.de/cat12/

2
http://www.restfmri.net
